# Can a Simple Dietary Screening in Early Pregnancy Identify Dietary Habits Associated with Gestational Diabetes?

**DOI:** 10.3390/nu11081868

**Published:** 2019-08-11

**Authors:** Laufey Hrolfsdottir, Ingibjorg Gunnarsdottir, Bryndis Eva Birgisdottir, Ingibjorg Th Hreidarsdottir, Alexander Kr. Smarason, Hildur Hardardottir, Thorhallur I. Halldorsson

**Affiliations:** 1 Unit for Nutrition Research, Landspitali University Hospital and Faculty of Food Science and Nutrition, University of Iceland, Eiríksgata 29 101 Reykjavik, Iceland; 2 Institution of Health Science Research, University of Akureyri and Akureyri Hospital, Eyrarlandsvegi, 600 Akureyri, Iceland; 3 Department of Obstetrics and Gynecology, Landspitali University Hospital, Hringbraut, 101 Reykjavík, Iceland; 4 Faculty of Medicine, University of Iceland, Vatnsmýrarvegi 16, 101 Reykjavík, Iceland; 5 Centre for Fetal Programming, Department of Epidemiology Research, Statens Serum Institut, Artillerivej 5, 2300 Copenhagen, Denmark

**Keywords:** dietary habits, maternal nutrition, gestational diabetes, food frequency questionnaire, dietary screening

## Abstract

Gestational diabetes mellitus (GDM) is predominantly a lifestyle disease, with diet being an important modifiable risk factor. A major obstacle for the prevention in clinical practice is the complexity of assessing diet. In a cohort of 1651 Icelandic women, this study examined whether a short 40-item dietary screening questionnaire administered in the 1st trimester could identify dietary habits associated with GDM. The dietary variables were aggregated into predefined binary factors reflecting inadequate or optimal intake and stepwise backward elimination was used to identify a reduced set of factors that best predicted GDM. Those binary factors were then aggregated into a risk score (range: 0–7), that was mostly characterised by frequent consumption of soft drinks, sweets, cookies, ice creams and processed meat. The women with poor dietary habits (score ≥ 5, *n* = 302), had a higher risk of GDM (RR = 1.38; 95%CI = 3, 85) compared with women with a more optimal diet (score ≤ 2, *n* = 407). In parallel, a pilot (*n* = 100) intervention was conducted among overweight and obese women examining the effect of internet-based personalized feedback on diet quality. Simple feedback was given in accordance with the answers provided in the screening questionnaire in 1st trimester. At the endpoint, the improvements in diet quality were observed by, as an example, soft drink consumption being reduced by ~1 L/week on average in the intervention group compared to the controls. Our results suggest that a simple dietary screening tool administered in the 1st trimester could identify dietary habits associated with GMD. This tool should be easy to use in a clinical setting, and with simple individualized feedback, improvements in diet may be achieved.

## 1. Introduction

Gestational diabetes mellitus (GDM) is a major pregnancy complication, defined as glucose intolerance with onset during pregnancy. The prevalence of GDM in European countries ranges from 2 to 22% with a median of 6% [1]. GDM has been associated with several adverse outcomes [2], including offspring macrosomia [3] and the mothers’ increased risk of the development of type 2 diabetes postpartum [4]. Longitudinal studies also suggest that offspring of mothers diagnosed with GDM are more prone to metabolic abnormalities later in life [5].

High pre-pregnancy BMI is one of the strongest risk factors for GDM and it is commonly used to identify pregnant women at risk for further monitoring [6]. The use of BMI to identify women at GDM risk does, however, have its limitations as high BMI alone is by no means an indicator for unhealthy dietary habits or a lifestyle that may influence GDM risk. Improvements in weight are also difficult to achieve in the short term and during pregnancy, other modifiable risk factors, such as diet, must be prioritised.

Recent observational studies indicate that unhealthy dietary habits before [7,8] and during pregnancy [9,10,11,12] are associated with a higher risk of GDM. Despite the observational nature, the link between poor carbohydrate and fat quality observed in these studies is both biologically plausible and in line with the established risk factors for type 2 diabetes [13]. However, an important limitation for targeting dietary habits of pregnant women is that the dietary assessment methods, developed for research purposes, are time-consuming and difficult to use in maternal care [14]. The complexity of diet is also challenging in terms of focusing efforts. To bypass this challenge, previous intervention studies have often focused on adherence to relatively strict dietary regimes aimed at achieving major changes [15,16,17]. These strategies have generally resulted in limited compliance and unclear benefits, highlighting the need to explore more targeted and flexible approaches.

The aim of this study was to examine if a short dietary screening questionnaire administered in weeks 11–14 of gestation could be used to identify unhealthy dietary habits associated with GMD and, in parallel, to investigate if a simple personalized web-based feedback tool could result in improvement in dietary habits in pregnancy.

## 2. Materials and Methods

This study was based on two studies of different designs that recruited pregnant women in Iceland from 2015 to 2017. In a cohort setting, this study examined, in a set of 1651 women, the association between dietary habits recorded in early pregnancy and the risk of GDM. In parallel, a pilot (*n* = 100) intervention study was conducted to test to what extent dietary changes could be achieved in the study population. In terms of the prevention and use in a clinical setting, an association between dietary habits and pregnancy complications such as GDM is limited on its own, unless there are indications that changes in those dietary factors can be modified using low intensity and cost-effective measures. This is the logic behind conducting these studies and to combine them in one paper.

### 2.1. The Cohort Study

The PREWICE cohort (PREgnant Women of ICEland) has been described elsewhere [18]. All women (*n* = 2734) with singleton pregnancies who attended a routine early ultrasound (11–14 week of gestation) at the prenatal diagnostic unit at Landspitali University Hospital in Reykjavik, South Iceland between 1 October, 2015 to 31 September, 2016, were invited to take part in the study. In total, 2113 women, (~50% of all births in Iceland during the study period) agreed to participate, whereof 417 (~20%) had missing hospital records, most likely as they gave birth outside Landspitali University Hospital. The additional 26 women who had multiple births and another 19 who had missing dietary data were excluded, resulting in 1651 women being eligible for analyses. No major differences were found in the baseline characteristics or dietary measures among those who were included in the full analysis and those who were excluded because of missing data [18]. 

#### 2.1.1. The Outcome Variable

The information regarding GDM cases was retrieved from maternal hospital records (ICD-10 codes O24.4 and O24.9, but O24.9 is used at Landspitali GDM treated with medications). The criteria for GDM diagnoses was according to the 2010 International Association of Diabetes and Pregnancy Study Groups (IADPSG) Consensus Panel [6].

#### 2.1.2. The Dietary Assessment and the Dietary Risk Score

**Figure 1:**
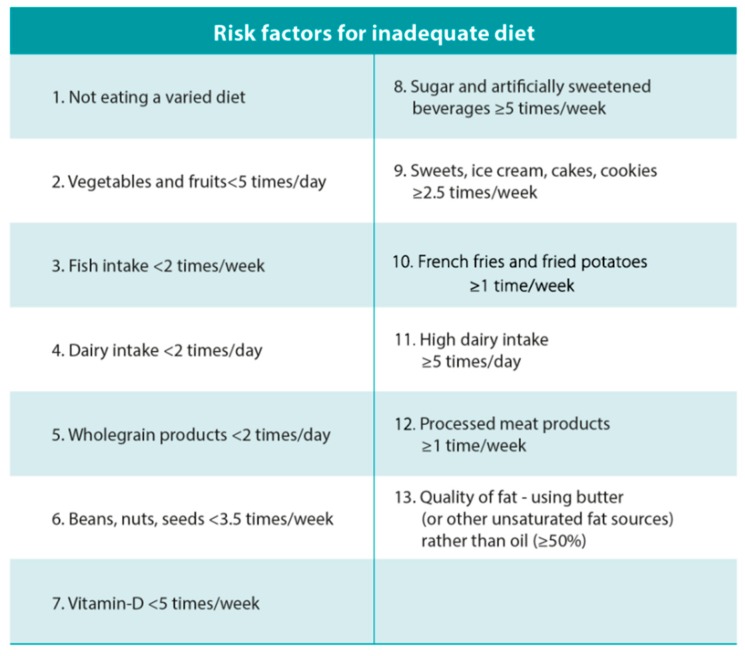
The predefined dietary risk factors for inadequate diet. The risk factors were mainly based on the Nordic Nutrition Recommendations [19] and the Icelandic Food-Based Dietary Recommendations [20]. If the women excluded/avoided any of the main food groups (cereal, vegetables/fruits, fish, meat, eggs, high-fat foods or dairy), they were categorized to the group of not eating a varied diet.

The details about the dietary assessment and construction of the dietary risk score have been described in detail elsewhere [18]. In short, the dietary screening questionnaire consisted of a 40-item list of common foods and food groups for which the frequency of consumption was recorded (see Supplemental File S1). The list was designed to give a rough overview of the participant’s general diet in comparison to current food-based dietary guidelines. The dietary data collected was converted to frequency per week for all food groups, which was then transformed into 13 predefined dietary risk factors for inadequate diet (Figure 1). The 13 factors used to construct the risk score are based on the Nordic [19] and Icelandic dietary recommendations [20] and supported by evidence from studies conducted in pregnant women [21,22,23,24,25].

Using these 13 predefined dietary risk factors as inputs, a stepwise backward elimination was used to identify a reduced set of variables with the highest maximum likelihood that best predicted GDM. The model performance was assessed by Nagelkerke’s R^2^. The following set of seven dietary risk factors (predictors) were included in the final model: Non-varied diet; sugar/artificially sweetened beverages ≥5 times/week; sweets, ice cream, cakes, cookies ≥2.5 times/week; processed meat products ≥1 time/week; whole grain products <2 times/day; dairy <2 times/day and vitamin D intake <5 times/week. The set of seven variables was then used to calculate a combined dietary risk score. Each participant got 1 score for fulfilling the risk criteria, and 0 for not fulfilling the risk criteria. The scores of the dietary risk factors were then summed up, ranging from 0 to 7.

To verify the the stability of our findings in terms of variable selection, comparable risk scores were created based on either a fewer or a larger number of dietary factors being retained in the model. The associations between these different scores in relation to GDM risk are shown in Table A1 (Appendix A).

### 2.2. The Pilot Intervention Study

For the pilot intervention study, 100 women with pre-pregnancy body mass ≥25 kg/m^2^, attending prenatal care at the Health Care Institution of North Iceland were recruited. At recruitment, i.e., at the first antenatal care visit, the women answered the same dietary questionnaire as described above for the cohort study. The participants were then randomized into a control group receiving the habitual care of brochures on the recommended diet during pregnancy according to clinical guidelines (*n* = 50; 9 dropouts), and an intervention group (*n* = 50; 3 dropouts), receiving personalized feedback on their diet quality through an interactive website [26]. This website was designed to give each woman simple personalized feedback, aimed at improving diet quality, in accordance with the answers provided by the dietary questionnaire. At baseline, a nutritionist showed the participants in the intervention group the website and went through the personalized feedback with them. Dietary intake in gestation weeks 24–26 and 35–38 was assessed by two 24-h recalls. These recalls were performed by another nutritionist who was blinded to the intervention assignment.

### 2.3. Statistical Analyses

The students t-tests were used to compare the normally distributed continuous variables, whereas, for skewed and categorical variables, the Mann-Whitney U test and Chi-square tests were used, respectively.

For the cohort study, the associations between the diet reported at baseline and GDM was assessed using multivariable Poisson log-linear regression. The covariates included in the adjusted models were selected, a priori, based on their potential influence on dietary habits and GDM [27,28,29,30,31]. The covariates included in the regression analyses were: Maternal pre-pregnancy BMI (<25, 25–29.99, ≥30 kg/m^2^); maternal age (quartiles); parity (nulliparous versus primi/multiparous); education (elementary schooling, high school and/or technical school, university education, and higher academic education); maternal smoking during pregnancy (yes/no) and family history of type 2 diabetes. The information on these covariates was recorded at recruitment with the exception of maternal age and family history of type 2 diabetes, which was retrieved from hospital records. The missing values for covariates (maternal pre-pregnancy BMI (0.7%), parity (1.2%), educational level (0.9%), and maternal smoking during pregnancy (1.5%) were assumed to be missing at random and were imputed using multiple imputations as implemented in *proc MI* in SAS v9.2 8. Statistical significance was accepted at *p* < 0.05.

### 2.4. Ethics

The ethics committee of Landspitali University Hospital approved the study protocol (21/2015) for the cohort study. For the intervention study, ethical approval was received from The National Bioethics committee (VSN-15-111-S1). Written consent was obtained from all participants in both studies.

## 3. Results

The characteristics of the PREWICE cohort are presented in Table 1. The mean age was 30 years, and most (94%) participants were non-smokers, 39% were nulliparous and 58% of the women had a university education or higher academic degree. In total, 16% (*n* = 265) were diagnosed with GDM during pregnancy. Stratified by pre-pregnancy weight, the prevalence of GDM among women of normal weight (BMI < 25 kg/m^2^), overweight (BMI 25–29.99 kg/m^2^) or obesity (BMI ≥ 30 kg/m^2^) was 5%, 15% and 49%, respectively. The mothers who developed GDM were also more likely to be older, Primi/multiparous and have a lower educational level (Table 1).

The number of women who fulfilled the risk criteria for each of the identified dietary risk factors is shown in Table 2. In total, 21% reported that they had a nonvaried diet, i.e., that they avoided or excluded some food groups. In total, 28% frequently consumed sugar and artificially sweetened beverages (≥5 times/week), 59% had a frequent intake of sweets, ice creams, cakes and cookies (≥2.5 times/week), and 31% ate processed meat products weekly (≥1 time/week). Most women did neither meet the public recommendations for whole grain intake of at least two portions per day (91%) nor the two recommended dairy portions per day (78%), and 30% of the women reported intake of vitamin D supplements less than <5 times per week. A higher proportion of women with GDM fulfilled the risk criteria for the consumption of sugar and artificially sweetened beverages (*p* ≤ 0.01) and processed meat products (*p* = 0.04) compared with women with no GDM. The other dietary risk factors did not differ significantly (Table 2).

In Table 3, the results for the multivariable association between the dietary risk score and GDM are presented. When dichotomizing the exposure, women with a high (≥5, *n* = 302) versus low (≤2, *n* = 407) dietary risk scores had 38% higher relative risk (RR) (95%CI: 3, 85%) of being diagnosed with GDM. The effect modification by pre-pregnancy BMI was formally tested. This was done by including the dietary risk score (continuous variable), BMI (continuous variable) and an interaction term between the two in the logistic regression model, along with the remaining covariates. An interaction was not observed (*p* = 0.81). In accordance to these results, similar results were found when comparing those with a high versus low dietary risk score among women with BMI < 25 (RR = 1.11 95%CI: 0.90, 1.36) vs. BMI ≥ 25 (RR = 1.09 95%CI: 1.002, 1.19).

In the pilot study, neither differences in the background variables (Table 4) nor dietary intake at baseline (Table A2) were observed. In total, 88 women completed both 24 h recalls (47 in the intervention group and 41 in the control group). Table 4 shows the median intake of selected food groups based on the two 24 h recalls. At the endpoint, soft drink consumption was significantly lower in the intervention group compared with the control group, corresponding to approximately one liter less consumption per week. Other differences in dietary intake between the groups were not statistically significant.

## 4. Discussion

This study found that a dietary risk score, partly characterized by frequent consumption of sugar/artificially sweetened beverages, sweets, cookies, ice creams and processed meat products, was associated with GDM diagnoses. The most pronounced differences in dietary habits of GDM versus non-GDM cases, as recorded at baseline, was excessive (≥5 times/week) consumption of soft drinks. The pilot intervention study, conducted in parallel, showed that internet-based personalized feedback on diet, reported early in pregnancy, could substantially reduce soft drink consumption. Whether such reduction can reduce GDM risks needs to be explored further in an intervention setting.

The results of this study are in line with previous studies using more detailed dietary assessment methods. For example, a recent Icelandic study showed that women who were overweight or obese but had a healthy diet were not at higher risk of gestational diabetes in comparison to women with normal weight [9]. Moreover, unhealthy dietary patterns, soft drinks and the intake of foods high in added sugar have previously been linked to a higher risk of GDM [10,11,12,32,33]. The regular intake of processed meat products has also commonly been associated with a higher risk of Type 2 diabetes [34,35] and GDM [8,12] and there are some indications that poor vitamin D status, commonly observed at Northern latitudes, may also be important for glucose homeostasis [36].

The Finnish Gestational Diabetes Prevention Study (RADIEL) [37] succeeded in reducing the overall incidence of GDM. However, here the focus was on obese women (and women with previous history of gestational diabetes) without taking into account the baseline dietary habits. Although high pre-pregnancy BMI status is a strong risk factor for GDM, not all overweight or obese women develop GDM [28] and as a substantial proportion of the general population is overweight and obese [38] more precise cost-effective risk assessment is needed.

Apart from GDM, a healthy diet in pregnancy has also been associated with a decreased risk for several other pregnancy and birth outcomes [23,24,39,40,41,42,43,44,45]. However, the translation and implementation of these results into clinical practice is still a challenge. One reason for this is that dietary assessment tools used in research settings are very time-consuming and not suitable for use in maternal care. The motivation for the studies reported here was to develop and test a diet screening questionnaire to be used in combination with a web-based platform that automatically gives users feedback on their diet during pregnancy [26]. This application is currently being tested for integration into the National Citizen Health Portal in Iceland. The portal is a centralized web-application where all citizens have secure, digital access to their own health information (e.g., maternal records) and official eHealth services currently available in the country. The inclusion of dietary screening into the portal may increase the feasibility of implementing dietary screening in early pregnancy on a national level and for use in clinical practice. To the authors’ best knowledge, information on dietary intake (other than supplement use) is not recorded anywhere in national prenatal health registries, but valid information about diet quality in early pregnancy might change the way women in need for dietary support during pregnancy are defined.

The main strengths of our cohort study include prospective data collection, high participation rates (77%), and information on GDM retrieved from medical records. However, as with all observational studies, the role of confounding cannot be excluded. It is important to note that the methodology used in our cohort study is based on both predefined and data-driven methods, tested in Iceland. The observed association with GDM might therefore not apply directly to other populations. However, this approach, i.e., to use a short FFQ (that takes into consideration population’s dietary habits) and to identify the potential risk factors based on existing recommendations, can be used in other settings to generate comparable (but perhaps not identical) results valuable for use in clinical practice. Using the same methodology as used in the present study, the authors have previously identified a dietary risk score that predicts the risk of excessive gestational weight gain and giving birth to a child weighing >4500 g (defined as macrosomia) independent of other known risk factors [18]. These findings need to be tested further in well-powered intervention settings. The short dietary screening questionnaire has been validated against 4-day weighed food records in a pilot study among 25 pregnant women (Spearman’s correlation >+0.3). The validation against biochemical analysis is part of an ongoing project (PREWICE II). Its ability to rank subjects according to consumption will be assessed, as well as its ability to detect the risk of predefined nutrient deficiency.

The pilot study had several limitations including a few participants. Moreover, the intervention consisted only of one contact with the participants at baseline. More frequent conversations about the results of the diet screening might have resulted in a greater difference between the groups [43,44]. However, the strength of this approach was its simplicity. This study showed that a simple intervention can result in dietary changes, i.e., lower soft drink consumption, in a population where excessive soft drink intake (≥5 times/week) was relatively common (~28%). While there was a trend towards improvement for other factors, no significant differences were observed. However, in the context of the result from the cohort study, these results are important as the most pronounced difference in the diet between GDM and non GDM cases was excessive consumption of soft drink (see Table 2). Previous intervention studies aimed at encouraging lifestyle changes to reduce the risk of GDM have often selected participants and tested interventions [46] regardless of the participants baseline diet and other lifestyle habits. The results from our studies here and recent work [47] suggest that a sensible way forward would be to select participants based on their baseline diet or other lifestyle factors that can be improved. Prioritizing and identifying a few factors to focus on might also be more attainable for women and more practicable in the clinical setting.

In summary, our results showed that a simple dietary screening tool administered in the 1st trimester could be used to identify dietary habits associated with GMD. This approach could be used to identify women in more need of support and diet counselling. Moreover, the results from the pilot intervention indicate that improvements in dietary quality can be achieved using low intensity and cost-effective measures. This procedure might strengthen preventative measures and enable targeted intervention among individuals most likely to benefit.

## Figures and Tables

**Table 1:** Birth outcomes and characteristics of mothers at baseline in relation to gestational diabetes mellitus diagnoses.

	All ^a^	GDM ^a,b^	No GDM ^a^	*p* Value ^c^
	(*n* = 1651)	(16%)	(84%)	
Maternal age (year)	30.3 ± 5.2	31.8 ± 5.4	30.0 ± 5.1	<0.01 ^d^
Height (cm)	167.5 ± 6.1	166.5 ± 6.3	167.7 ± 6.1	<0.01 ^d^
Birth weight (g)	3670 ± 552	3686 ± 587	3667± 545	0.64 ^d^
Gestational age (weeks)	40.0 (1.0)	39.0 ± 2.0	40.0 ± 2.0	<0.01 ^e^
Pre-pregnancy weight (kg)	68.0 (20.0)	85.0 (19.5)	66.0 (16.0)	<0.01 ^e^
Pre-pregnancy BMI (kg/m^2^)	24.2 (6.7)	30.5 (7.6)	23.5 (5.3)	<0.01 ^e^
Pre-pregnancy BMI (groups)				<0.01 ^f^
<18.5 kg/m^2^ (%)	3	1	4	
18.5–24.99 kg/m^2^ (%)	54	17	61	
25–29.99 kg/m^2^ (%)	24	24	23	
≥30 kg/m^2^ (%)	19	59	11	
Exc. GWG (%)	36	33	36	0.38 ^f^
Parity (%)				<0.01 ^f^
Nulliparous	39	31	41	
Primi/multiparous	61	69	59	
Single (%)	6	7	5	0.34 ^f^
Smoking during pregnancy (%)	6	7	6	0.74 ^f^
Family history of type 2 diabetes (%)	18	15	39	<0.01 ^f^
Education (%)				<0.01 ^f^
Elementary schooling	13	18	12	
High sch. and technical sch.	30	32	29	
University education	34	37	34	
Higher academic	24	14	26	

Abbreviations: BMI, body mass index; GDM, gestational diabetes mellitus; GWG, gestational weight gain. ^a^ Values are mean ± standard deviation or median (IQR) for continuous variables and percentages for categorical variables; ^b^ The criteria that was used [6] ^c^ Differences between GDM and no GDM. ^d^ F-test (Type III) of differences among groups. ^e^ Mann-Whitney U test of differences among groups. ^f^ Chi-square test of differences among groups.

**Table 2:** Percent of women fulfilling the predefined risk criteria.

Risk factors	All (*n* = 1651)	GDM ^a^ (16%)	No GDM (84%)	*p* ^b^
Not eating a varied diet	21%	23%	21%	0.43
Sugar and artificially sweetened beverages ≥5 times/week	28%	37%	27%	<0.01
Sweet, ice cream, cakes, cookies ≥2.5 times/week	59%	63%	58%	0.14
Processed meat products ≥1 time/week	31%	37%	30%	0.04
Whole grain products <2 times/day	91%	93%	91%	0.26
Dairy <2 times/day	78%	81%	77%	0.18
Vitamin D intake <5 times/week	30%	34%	29%	0.12

^a^ The criteria that was used [6]. ^b^ Chi-square test of differences among groups (GDM vs. no GDM).

**Table 3:** The association between the dietary risk score and gestational diabetes ^a^.

		RR (95% CI) ^b^	
	Cases (%)/*n*	Crude	Adjusted ^c^
Dichotomized score			
≤2 scores	49 (12%)/407	ref	ref
3 scores	76 (15%)/503	1.26 (0.90, 1.75)	0.96 (0.72, 1.30)
4 scores	69 (16%)/439	1.31 (0.93, 1.83)	1.00 (0.74, 1.37)
≥5 scores	71 (24%)/302	1.95 (1.40, 2.72)	1.38 (1.03, 1.85)
*p* for trend		<0.01	0.02
Stratified analyses, continuous score			
All women	265 (16%)/1651	1.20 (1.10, 1.32)	1.10 (1.02, 1.20)
BMI < 25 kg/m^2 d^	51 (5%)/947	1.20 (0.96, 1.52)	1.11 (0.90, 1.36)
BMI ≥ 25 kg/m^2 d^	214 (30%)/704	1.12 (1.02, 1.23)	1.09 (1.002, 1.19)

Abbreviations: BMI, body mass index; ^a^ The criteria that was used [6]. ^b^ Logistic regression model reflecting the odds of GDM. ^c^ Adjusted for maternal pre-pregnancy BMI, age, parity, smoking during pregnancy, educational level and family history of type 2 diabetes. ^d^ Pre-pregnancy BMI not included as a covariate.

**Table 4:** Baseline characteristics and dietary habits at the endpoint among pregnant women participating in the pilot intervention.

	Control (*n* = 41) ^a^	Intervention (*n* = 47) ^a^	*p*-Value ^b^
Baseline Characteristics			
Pre-pregnancy BMI (kg/m^2^)	28.7 (27.1–31.5)	29.4 (27.5–35.2)	0.40
Gestational length at baseline (weeks)	15.0 ± 2.5	14.8 ± 2.7	0.83
Age			0.27
18–24, *n* (%)	10 (24)	10 (21)	
25–34, *n* (%)	28 (68)	28 (60)	
≥35, *n* (%)	3 (7)	9 (19)	
Parity			0.27
Nulliparous, *n* (%)	13 (32)	10 (21)	
Primi/multiparous, *n* (%)	28 (68)	37 (79)	
Smoking during pregnancy, *n* (%)	5 (12)	5 (11)	0.82
Dietary habits at endpoint ^c^			
Milk and cultured milk products (g/d)	217 (138–396)	247 (102–376)	0.91
Vegetables (g/d)	91 (23–148)	101 (57–135)	0.18
Fruits and berries (g/d)	105 (73–220)	150 (70–215)	0.67
Fish ≥300g/week (%)	22%	32%	0.30
Processed meat (g/d)	14 (0–42)	10 (0–25)	0.69
Soft drinks (g/d)	125 (25–365)	75 (0–200)	0.03
French fries or chips ≥100g/week (%)	27%	19%	0.38
Cakes, biscuits, and/or sweets (g/d)	62 (19–114)	37 (18–88)	0.25

^a^ Values are mean ± standard deviation or median (25th–75th centiles) for continuous variables and percentages for categorical variables. ^b^ F test (Type III) or Mann-Whitney U test was used to assess differences among groups for continuous variables and Chi-square test for categorical variables. ^c^ Mean of two 24 h recalls in gestational weeks 24–26. and 35–38.

## Supplementary Materials

The following are available online at https://www.mdpi.com/2072-6643/11/8/1868/s1, File S1: The dietary screening questionnaire (English translation).

## Appendix A

As the use of backward elimination for selecting factors that predict gestational diabetes involves some arbitrary decisions in terms of where to stop the elimination process, we examined the stability of our findings by creating standardized risk scores based on fewer and more dietary factors being retained in the model (Table A1).

SCORE-1 included two dietary risk factors, i.e., the factors most strongly associated with GDM in the multivariable model: sugar and artificially sweetened beverages ≥5 times/week and processed meat products ≥1 time/week.

SCORE-2 included seven dietary risk factors, i.e., a non-varied diet, sugar and artificially sweetened beverages ≥5 times/week, sweet, ice cream, cakes, cookies ≥2.5 times/week, processed meat products ≥1 time/week, whole grain products <2 times/day, dairy <2 times/day and vitamin D intake <5 times/week.

SCORE-3 included all 13 dietary risk factors, i.e., not eating a varied diet, vegetables and fruits <5 times/day, fish intake <2 times/day, dairy intake <2 times/day, whole grain products <2 times /day, beans, nuts, seeds <3.5 times/week, vitamin D <5 times/week, quality of fat—using butter rather than oil (≥50%), french fries and fried potatoes ≥1 time/week, sweets, ice cream, cakes, cookies ≥2.5 times/week, sugar and artificially sweetened beverages ≥5 times/week, dairy intake ≥5 times/day, processed meat products ≥1 time/week.

As can be seen in Table A1, The combination of the seven dietary risk factors resulted in the most significant association.

**Table A1:** Different combinations of the dietary risk score and the risk of gestational diabetes mellitus (GDM) ^a^.

	Crude ^b^ RR (95%CI)	Adjusted ^b,c^ RR (95%CI)
SCORE-1	1.23 (1.10, 1.36)	1.11 (1.00, 1.22)
SCORE-2	1.26 (1.13, 1.40)	1.13 (1.02, 1.25)
SCORE-3	1.22 (1.09, 1.36)	1.10 (0.99, 1.22)

^a^ The criteria that was used [6]. ^b^ Standardized coefficient reflecting the risk of GDM per standard deviation increase in the dietary risk score. ^c^ Adjusted for maternal pre-pregnancy BMI, age, parity, smoking during pregnancy, educational level and family history of type 2 diabetes.

**Table A2:** Baseline dietary intake (frequency per week) of subjects in the pilot study.

5	Control (*n* = 41) ^a^	Intervention (*n* = 47) ^a^	*p* Value ^b^
Milk	5.3 (2.2–7.6)	5.4 (2.1–14.4)	0.289
Cultured milk	5.0 (1.0–5.0)	2.5 (1.0–5.0)	0.876
Beans, nuts and/or seeds	0.5 (0.1–1.0)	0.5 (0.1–2.5)	0.244
Vegetables	7.0 (3.8–7.0)	7.0 (2.5–14)	0.202
Fruits and berries	14.0 (7.0–14)	7.0 (5.0–14)	0.655
Fish	1.1 (1.0–2.8)	1.5 (0.8–2.8)	0.803
Processed meat	0.5 (0.3–0.8)	0.5 (0.3–1.0)	0.158
Soft drinks	2.0 (0.6–5.0)	2.0 (0.6–3.5)	0.556
French fries or chips	0.5 (0.5–1.0)	0.5 (0.5–1.0)	0.554
Cakes, biscuits, ice and sweets	3.5 (1.5–6.0)	2.7 (1.5–5.0)	0.058

^a^ Values are median (25th–75th centiles). ^b^ Mann-Whitney U test was used to assess differences among groups.
