# Supplementary material for: Can a Simple Dietary Screening in Early Pregnancy Identify Dietary Habits Associated with Gestational Diabetes?
Source: Nutrients. 2019 Aug 11;11(8):1868. doi: 10.3390/nu11081868 (PMC6722606; doi:10.3390/nu11081868)
Supplement: Supplementary file 1 [file nutrients-11-01868-s001.pdf]

HÁSKÓLI ÍSLANDS

Number:

FOOD SELECTION

Do you avoid or not eat certain types of food? **Yes ___** **No ____**

If yes, mark the types of food that you avoid or do not eat.

**Cereal products**	
**Vegetables**	
**Fruits**	
**Fish**	
**Meat**	
**Eggs**	
**High-fat foods**	
**Dairy products**	

CONSUMPTION OF VARIOUS FOODS

Keeping in mind the last 4 weeks, put one cross in each row.

How many times per month or week or day do you eat the following types of food?
(Questions on fish and meat (including processed meat products) apply to main meals, not sandwich meats or spreads.)

	Per month			Per week			Per day			
	<1	1	2-3	1	2-3	4-6	1	2	3-4	≥5
Vegetables										
Fruits										
Lean fish (e.g., haddock or cod)										
Fatty fish (e.g., salmon, trout or large halibut)										
Red meat (beef, lamb or pork)										
Poultry										
Processed meat products, meat dough products or sausages										
Soured dairy products (sour milk, skyr or yogurt)										
Cheese										
Whole-grain products, other than bread*										
Bean dishes, nuts or seeds (not in breads)										
French fries and/or packaged snacks										
Cakes and/or crackers										
Candy and/or ice cream										

*E.g., brown rice, barley or whole-wheat pasta, as accompaniment or part of main meals.

HIGH-FAT FOODS

Keeping in mind the last 4 weeks, put one cross on each row.

How often do you use the following high-fat foods?

	Per month			Per week			Per day			
	<1	1	2-3	1	2-3	4-6	1	2	3-4	≥5
Oil or other soft fat for food preparation										
Butter or other hard fat for food preparation										
Butter or oil-blended butter on bread										

BEVERAGES

Keeping in mind the last 4 weeks, put one cross on each row.

How many portions per month or per week or per day do you drink of the following beverages?

Assume that one portion is about 250 ml.

Remember to include in the estimate milk on morning cereal or mush and in coffee. Carbonated or noncarbonated beverages include all types of carbonated beverages, sports drink, fruit drink (other than pure fruit juices) and energy drink.

	Per month			Per week			Per day			
	<1	1	2-3	1	2-3	4-6	1	2	3-4	≥5
Pure fruit juice										
Whole milk										
Low-fat milk										
Low-fat milk fortified with vitamin D										
Fortified nonfat milk (vitamin D- and protein-fortified)										
Nonfat milk										
Carbonated and noncarbonated drinks with added sugar										
Carbonated and noncarbonated drinks with sweeteners										
Coffee										
Alcohol										

HÁSKÓLI ÍSLANDS

BREAD

Keeping in mind the last 4 weeks, put one cross on each row.

How many slices per month or per week or per day do you eat of the following kinds of bread?

	Per month			Per week			Per day			
	<1	1	2-3	1	2-3	4-6	1	2	3-4	≥5
**Whole-grain bread.** This means breads marked with the Keyhole, or specified as fiber rich or whole grained.										
**Other breads.** This means "usual" wheat breads (Heimilisbrauð, Bónus/Krónu-brauð, ciabatta, soft-cheese bows, etc.)										
**Rye bread with added sugar.** Here, this pertains only to Icelandic rye bread. Please record other rye bread, e.g., Danish rye bread, as whole-grained bread.										

SUPPLEMENTS

Keeping in mind the last 4 weeks, put one cross on each row.

How often per month or per week or per day do you use the following supplements?

	Per month			Per week			Per day			
	<1	1	2-3	1	2-3	4-6	1	2	3-4	≥5
Cod liver oil										
Vitamin D										
Folate/folacin/folic acid										
Iron										
Multivitamins with vitamin A										
Multivitamins without vitamin A										
Other supplements or food additives*										

*Other supplements, which ones? ____

Thank you for participating!
